# Comparative analysis of sleep patterns and attention components in high school and college adolescents

**DOI:** 10.5935/1984-0063.20200085

**Published:** 2021

**Authors:** Fernanda Mayara Crispim Diogo, Sabinne Danielle Galina, Maria Luiza Cruz de Oliveira, Pablo Valdez, Carolina Virginia Macêdo de Azevedo

**Affiliations:** 1 Universidade Federal do Rio Grande do Norte, Department of Physiology and Behavior - Natal - RN - Brazil.; 2 Universidad Autónoma de Nuevo León, Departamento de Psicofisiologia -Monterrey - Nuevo León - Mexico.

**Keywords:** Sleep Deprivation, Sleepiness, Cognition, Students, Schools

## Abstract

Adolescence is a phase with physiological and behavioral changes. One of them occurs in the sleep-wake cycle pattern, manifested by a phase delay. However, morning school start time can decrease sleep duration during weekdays, impairing adolescent cognitive performance and well-being. Adolescents of different ages and educational level might suffer the impact of academic demand on sleep-wake cycle and cognition differently. Thus, the aim of this study is to compare the sleep habits and quality, sleepiness upon awakening and attention components among adolescents in the first years of high school and college. 71 adolescents participated in the study (45 girls and 26 boys), 44 enrolled in high school morning classes (G1 - 15.5±0.7 years), from a private school, and 27 college students enrolled in morning classes (G2 - 18.8±1.04 years), from biosciences courses from a public institution. The groups did not differ in bedtime, get up time, time in bed and sleep irregularity. However, both groups showed differences according to the day of the week, bedtime and get up time became later and time in bed extended on weekends. G1 presented worse sleep quality and regarding attention, showed higher percentage of omissions in all components and worse performance in sustained attention (ANOVA, p<0.05). The poorer sleep quality of high school adolescents and reduced attention may have a negative effect on school performance. Additional studies are needed to investigate the causes of these differences between these two educational levels.

## INTRODUCTION

Adolescents undergo several physiological and behavioral changes with puberty onset. One of these changes is observed in the sleep-wake cycle pattern, which is manifested by a phase delay, when bedtime and wake up time occurs later. This variation in sleep-wake cycle occurs as a consequence of modifications in the mechanisms that regulate sleep and in the psychosocial context^1,2^. The literature suggests a relation between changes in adolescent sleep patterns and the onset and course of puberty, which is the phase delay is more significant with maturation. Thus, older adolescents present a greater delay in the sleep-wake cycle pattern^2,3^.

The sleep-wake cycle is regulated by intrinsic brain mechanisms, described as the two-process model by Borbély et al. (2005)^5,6,7^. 1 - The circadian system (Process C) regulates several biological rhythms of approximately 24h duration. These rhythms are synchronized mainly by the light/dark and social cycles, and generated by a timing system, in which the suprachiasmatic nucleus (SCN) is appointed as the main pacemaker^6^. 2 - The homeostatic process (Process S) represents the sleep pressure in function of wakefulness and sleep duration, in which pressure to sleep increases during individual’s wakefulness phase and dissipates during sleep. In adolescence, the rate of homeostatic pressure build-up slows down, allowing them to stay awake longer and, consequently, to increase the exposure to light at night, delaying the circadian rhythm^2,9^.

Another aspect that changes in adolescence is chronotype, which is the individual’s diurnal preference that shape the timing of sleep hours and behavior. It is classified in 3 types, early (morning), intermediate and late (evening)^8,9^. The literature has shown that adolescents have a tendency to be evening types what impact the sleep-wake cycle and individual’s performance^10,11^. Evening type individuals may have poor sleep efficiency and quality, irregular sleep time and shorter sleep duration during weekdays^11,12^.

The psychosocial context also contributes to adolescents’ sleep-wake cycle, possibly because of the autonomy gain and the increase of academic demand^2,13^ and electronic media use^14^. In addition, the use of electronic media at night may promote suppression of melatonin secretion and a delay in circadian rhythms caused by blue light emission from these electronics devices^3,13^, as well as an increase on physiological arousal caused by its use^15^, causing more excitatory stimuli to the central nervous system at night.

In contrast, the morning school start time reduces the sleep duration leading to sleep deprivation and irregularity of sleep^2^. These factors can bring several consequences such as daytime sleepiness, mood disorders, behavioral problems, weight gain, impaired immune function^2,15^, alcohol and cigarette consumption^17^ and detrimental effects on cognition, especially attention^18^.

Attention is an essential cognitive process for various performance tasks^19^. According to Rafal and Posner (1987)^20^ model, modified by Valdez et al. (2005)^19^, attention can be defined by four components: 1 - tonic alertness, which is the ability to respond to the environment at any time; 2 - phasic alertness, which is the ability to respond to a stimulus after an alert signal; 3 - selective attention, which allows the individual to respond to a specific stimulus while ignoring others; and 4 -vigilance (sustained attention), which is the ability to maintain attention for a period of time^18,19,20^.

The literature supports the idea that sleep deprivation and/or irregular sleep hours impairs school performance. Adolescents who report shorter sleep times have the worst school performance than those who had longer sleep times^11,21^. In other studies, sleep irregularity was associated with lower cognitive performance and academic achievement in high school students^23^ and negatively correlated with academic performance in undergraduates^24^. Therefore, the sleep habits may have an impact on adolescents’ academic life and academic demands may influence sleep need affecting quality and duration of sleep. Thus, bad sleep habits may lead to poor school performance, with a detrimental effect on mental and physical health as well.

High school and college adolescents have different biological and psychosocial contexts. High school students have strict and fixed morning schedules, whereas undergraduates have a greater delay on the sleep-wake cycle and more flexibility to make their own schedules and autonomy with the sleep hours when compared to younger adolescents. Therefore, comparing the sleep patterns of these adolescents on different educational levels and age could enlighten if older adolescents experience more sleep deprivation and/or if the psychosocial context is more detrimental to adolescent’s sleep pattern and school performance. The results could also serve as a way of increase awareness to schools, parents and adolescents helping them to adopt measures to improve the sleep habits according to the psychosocial context.

The aim of this study was to compare sleep habits and quality, sleepiness upon awakening and attention components among adolescents in the first years of high school and college. We hypothesized that: 1 - sleep patterns, regardless of school level, vary between school-days and days off; 2 - the quality, duration and irregularity of sleep, sleepiness upon awakening and attention of college adolescents vary when compared to high school students.

## MATERIAL AND METHODS

### Participants

The participants were 71 adolescents who have morning classes at school in Natal/RN. The students were divided into two groups: a) Group 1 - 44 students (27 girls) from freshman and sophomore years in a private high school, with mean age of 15.5±0.7 years and school start time at 7:15 a.m. for everyone; and b) Group 2 - 27 students (18 girls) from the first years of college in the area of biosciences of a public institution, with mean age of 18.8±1.04 years and school start time varying between 7 and 10 a.m. (8 a.m.±1:00h) with differences among individuals as well as in each day along the week.

Participants who reported neurological and/or sleep disorder, visual impairment that could compromise performance of the attention task or the use of any medication that could affect individual’s performance and sensory perception were excluded from the sample. The study was approved by the Research Ethics Committee of Universidade Federal do Rio Grande do Norte (Protocols CEP No. 1.489.057 and 2.165.576).

### Protocol

To characterize the sample and apply the exclusion criteria, the students answered the “health and sleep” questionnaire^25^, which covers questions about sleep habits, general health, self-reported school performance, and extracurricular activities (physical activity and courses outside of school). Besides, the sleep quality was assessed by the Pittsburgh sleep quality index (PSQI). The PSQI may have an overall score of 0 to 21 points and a 5 point score or above indicates poor sleep quality^26^.

In addition, they filled out a sleep diary for 10 days, recording the sleep habits (bedtime, get up time, ways of awakening – if they woke up with the aid of alarm clocks, by someone else or spontaneously –, bedtime and get up reasons and nap frequency). For bedtime and get up time reasons, the participant would sign the boxes corresponding to what they were doing before going to bed and what was the reason that they had to wake up in that hour. The alternatives for bedtime reasons were: TV; PC; games; studying; working; partying; cell phone and others. For get up time reasons, the alternatives were: school; physical activity; trips; religious activity; to go out and others. Besides, the sleepiness upon awakening was evaluated by the Maldonado sleepiness scale with illustrative faces adapted by Belísio (2014)^27^, present in the diary. This scale varies from 1 to 9, with 9 being the highest level of sleepiness upon awakening.

Regarding attention components, the students performed a continuous performance task (CPT)^19^ between Tuesdays and Fridays, from 7 a.m. to 10 a.m. A CPT version^19^ was applied through a computer. The version consists of 27 blocks, each with 20 numerical stimuli, 540 stimuli in total, duration of 11 minutes and 42 seconds. Volunteers were asked to use their index, middle, and ring fingers to press the numbers 1, 2, and 3 on the computer keyboard, respectively, according to the number on the screen. The number sequence varied from 0 to 9, number 1 should be pressed when the number on the screen belonged to 0 to 8 sequence (tonic alertness); 2 should be pressed only when number 9 appeared on the screen (selective attention); and number 3 when 4 appeared after 9 (phasic alertness). Sustained attention was assessed by the individual ability to focus throughout the task.

### Data analysis

The high school and college students formed two study groups named G1 and G2, respectively. The sleep habits (bedtime and get up time, time in bed, and the duration of naps) were compared between the two groups by *factorial ANOVA*, using as factors *group* and *day of the week*. Three indices were calculated to characterize sleep irregularity: bedtime irregularity (calculated from the differences in the means of bedtimes between weekend and weekdays), the get up time irregularity (calculated from the differences in the means of get up times between weekend and weekdays) and the time in bed irregularity (calculated from the differences in the means of time in bed between weekend and weekdays). These three indices, the scores of sleep quality, the sleepiness upon awakening and the self-reported school performance were compared between groups using *One way ANOVA*. The reasons to bed and get up time, the ways of awakening, the extracurricular activities and the proportion of PSQI classification (percentage of individuals with poor and good sleep quality) were analyzed using chi-square test.

Regarding the performance in CPT, the following variables were considered for each component of attention: 1 - the reaction time of the correct responses; 2 - the percentage of correct responses; and 3 - the omissions, calculated from stimuli not answered by the participants. The sustained attention was evaluated by the general stability (standard deviation of reaction time of correct responses), in that, lower standard deviation indicates high stability, i.e. good sustained. All attention variables were compared between groups using ANOVA. The level of significance of 5% was considered for all tests. Besides, p-values between 0.05 and 0.10 were considered as a tendency to significance.

## RESULTS

### Sleep habits

There was no difference between groups in relation to bedtime (F_(1,702)_=0.23, *p*=0.62 ; Tukey, *p* > 0.05 - Table 1) and get up time (F_(1,702)_=0.06, *p*=< 0.79; Tukey, *p* > 0.05), irrespective of the day of week. Both groups went to bed and got up later on weekend (ANOVA, *p*<0.05). Besides, the groups showed no differences in time in bed during week and weekend days (F_(1,69)_=0.82, *p*=0.36; Tukey, *p* > 0.05). Both groups stayed in bed longer on weekends (F_(1,69)_=75.38, *p*<0.05). There was no difference between groups in irregularities of bedtime, get up time and time in bed (ANOVA, *p*>0.05).

**Table 1:** Means and standard deviations of bedtime and get up time, time in bed, sleep irregularity, naps duration, PSQI score and percentage, and sleepiness upon awakening score according to group and day of the week.

Parameters	G1	G2	p (groups)	p (days)
**Bedtime**				<0.05
Weekdays	23h09±1.3	23h04±2.6	=0.97	
Weekend	00h09±1.7	00h05±1.5	=0.99	
**Get up time**				<0.05
Weekdays	06h05±0.8	06h22±1.8	=0.31	
Weekend	08h37±1.9	08h16±1.9	=0.30	
**Time in bed**				<0.05
Weekdays	06h57±1.4	07h06±2.1	=0.82	
Weekend	08h21±1.9	08h14±1.9	=0.96	
**Irregularity**				
Bedtime	1h16±0.5	0h59±1.4	=0.21	
Get up time	1h54±0.6	1h46±1.5	=0.99	
Time in bed	1h28±1.5	1h07±1.5	=0.31	
**Naps duration**				
Weekdays	01h19±0.9	01h26±0.9	=0.93	
Weekend	01h36±1.3	01h40±1	=0.99	
**PSQI Score**				
	11.6±5.9	6.9±1.8		<0.05
**PSQI %**				
Good	17.86%	30.56%		<0.05
Bad	82.14%	69.44%		
**Sleepiness upon awakening**				
Weekdays	4.7±2.3	4.8±1.9	=0.99	
Weekend	4.4±2.3	3.9±2	=0.79	

The groups did not differ in the proportion (weekdays: G1=25.5%; G2=26.21%; X^2^=0.07, *p*=0.78; weekend: G1=21.38%; G2=28.43%; X^2^=2.44, *p*=0.11) and in the duration of naps during weekdays and weekend (ANOVA, *p*>0.05 - Table 1).

### Bedtime and get up time reasons

**Figure 1:**
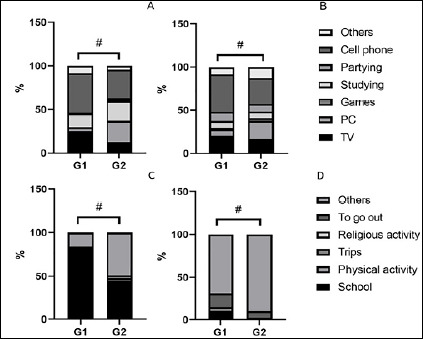
Group proportions of reasons for: bedtimes on A) weekdays and B) weekend; and for get up times on C) weekdays and D) weekend. #X^2^=p<0.05

The groups differed in what they did before bed during weekdays (X^2^=37.8, *p*<0.05 - Figure 1A). G1 had a higher proportion in use of cellphone, TV and PC, whereas G2 had a higher proportion of studying. A similar result was observed in weekend, with an inversion in the PC use that was higher in G2 (X^2^=18.6, *p*<0.05 - Figure 1B).

Get up time reasons during weekdays were different between groups (X^2^=53.5, *p*<0.05 - Figure 1C). Both groups had school as main reason; however, the proportion was higher in G1. During weekend, G1 still had a higher percentage for school than G2, which had other activities as major reason (X^2^=50.18, *p*<0.05 - Figure 1D).

### Ways of awakening

The groups differed in the ways of awakening during weekdays (X^2^=48.8, *p*<0.05). G1 showed a higher use of alarm clock (G1: 58.5%; G2: 55.6%) and being woke by someone else (G1: 32.7%; G2: 12.6%), while G2 showed a higher percentage of spontaneous awakenings (G1: 8.8%; G2: 31.8%). On weekend, G1 continued to need the alarm clock (G1: 28.1%; G2: 16.3%) and being woke by someone else (G1: 19.5%; G2: 16.3%), but the percentage of spontaneous awakenings increased, even though it remained less than G2 (G1: 52.4%; G2: 67.4% - X^2^=12.2, *p*<0.05).

### School performance and extracurricular activities

G1 reported worse school performance (G1=6; G2=7 - F_(1,74)_=11.1 *p*<0.05). Regarding the use of electronic media, G1 has more participants with TV in the bedroom (G1: 65.96%, G2: 17.86% - X^2^=27.8, *p*<0.05), however there was no difference in the amount of participants who have computer or laptop in the bedroom (G1: 65.96%, G2: 68.97% - X^2^=0.4, *p*=0.51). In relation to extracurricular activities, G1 does more physical activities (G1: 66.6%; G2: 43.3%; X^2^=22.1, *p*<0.05) and out-of-school courses (G1: 56.2%; G2: 10%; X^2^=237.6, *p*<0.05) when compared to G2.

### Sleep quality and sleepiness

Most participants in both groups had poor sleep quality, but G1 showed a higher percentage of students with poor sleep quality (G1: 82.14%; G2: 69.44% - X^2^=7.60, *p*<0.05 - Table 1) and a worse mean score of sleep quality (F_(1.79)_=15.2, *p*<0.05 -Table 1). Regarding sleepiness upon awakening, there was no difference between groups (F_(1,692)_=0.31, *p*=0.57 - Table 1), as well as between weekdays and weekend (F_(1.692)_=2.5, *p*=0.11).

### Attention

**Figure 2:**
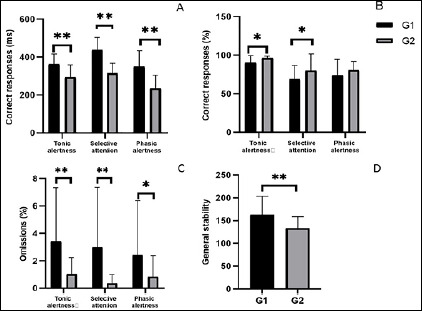
Variables related to components of attention of groups (means ± SD): A) Reaction time of correct responses; B) Percentage of correct responses; C) Percentage of omissions; and D) General stability (standard deviation of reaction time).

The groups differed in relation to reaction time of correct responses, G1 had a longer time for tonic alertness (F_(1,63)_=20.5, *p*<0.05), for selective attention (F_(1,62)_=54.1, *p*<0.05) and for phasic alertness (F_(1,63)_=28.1, *p*<0.05 -Figure 2A). Regarding the percentage of correct responses, G1 had a lower percentage than G2 in tonic alertness (F_(1,63)_=8.4, *p*<0.05), selective attention (F_(1,63)_=4.7, *p*<0.05), however there was no difference in phasic alertness (F_(1,63)_=1.8, *p*>0.05 -Figure 2B). In relation to percentage of omissions, G1 had a higher percentage in tonic alertness (F_(1,63)_=7.0, *p*<0.05), in selective attention (F_(1,63)_=7.5, *p*<0.05) and showed a tendency in phasic alertness (F_(1,63)_=3.1, *p*=0.08 - Figure 2C). Regarding the sustained attention, the groups differed in relation to general stability, G1 showed a higher standard deviation of reaction time (F_(1,63)_=9.1, *p*<0.05 - Figure 2D), indicating a lower general stability during the task.

## DISCUSSION

The aim of this study was to compare the sleep habits and quality, sleepiness upon awakening and performance in the morning in a task that assesses attention between adolescents in the first years of high school and college.

In line with the first hypothesis, it was observed that the sleep pattern of adolescents differed between schooldays and days off, when bedtime and get up time are later, regardless of the level of education. This reinforces the role of morning school start time as a social modulator for adolescent students irrespective of educational level. Similar results were found in the literature about high school^3,23^ and college adolescents^25,26^. Although, college students had higher variability in school start times along the week, an impact of this schedule on sleep patterns was not observed in our results. Future studies with a larger sample are necessary to clarify this possible effect.

According to the National Sleep Foundation, adolescents usually need to sleep between 8 and 10 hours by night in order to perform daily activities well^27^. However, the adolescents of this sample are sleeping less than 8 hours by night during schooldays, suggesting that these students are suffering from sleep deprivation, irrespective of school level. Time in bed was longer on days off for both groups, a pattern found in other studies^3,23,25,26^. This pattern is characterized by a decrease in time in bed during schooldays and increasing on days off as a way to compensate sleep deprivation. This sleep restriction-extension pattern is associated with irregular sleep times for adolescents of many countries^3,15,26^.

Regarding the sleep habits, the groups did not differ in relation to bedtime, get up time, time in bed and sleep irregularity, neither in naps frequency nor in sleepiness upon awakening. These results showed that the two levels of education have similar sleep habits. However, the sleep pattern of high school students is followed by higher reports of the need of alarm clocks and someone else to wake up, while for college students predominate the spontaneous awakening. These results might be related with poor sleep quality experienced by high school students, which can make it difficult to wake up spontaneously.

Most students had poor sleep quality. The literature shows that it is a common feature for adolescents, which may be related to the morning school start times, as well as the use of electronic media at night^27,28,29^. In our results, high school students had higher percentage of individuals with poor sleep quality and had the worst mean score of PSQI, which may be a consequence of more use of electronic media before bedtime observed in this group, damaging sleep quality. Besides, this might be associated to the direct impact of electronic media use suppressing melatonin secretion and increasing physiological arousal in response to the light emitted^13,14^. Other factors that were not analyzed in this study could be also negatively impacting the sleep quality of these students such as academic demand, mood and sleep disorders, especially considering that high school students are preparing themselves for exams to enter college. Thus, high school students could be experiencing a pressure by the school and parents to get admitted into a good college, which could lead to an increase of anxiety levels, impairing their sleep quality^30^.

Other studies observed that increased sleepiness would lead to poorer performance on cognitive tasks^18,31^. Regarding attention, almost all of the components showed differences between groups, high school students had the longest reaction time of correct responses for tonic alertness, selective attention and phasic alertness, and the lowest percentage of correct responses in tonic alertness and selective attention. High school students also had higher percentage of omissions in phasic alertness, selective attention and a tendency in phasic alertness and worse performance in general stability, indicating worse sustained attention. Our results did not show differences between groups in relation to sleepiness upon awakening, nevertheless, high school students showed the worst sleep quality score, which could explain the worst performance in CPT.

Tonic alertness is the responsiveness to the environment at any time^19^ and is related to the ascending reticular activating system^32^, which may be linked to the homeostatic component of sleep. Thus, sleep deprivation and poor quality can interfere with this component of attention. Therefore, it was expected that high school students had the worst performance for tonic alertness for having the worst sleep quality score and this was shown by the longest reaction time and lower percentage of correct responses for this component of attention.

Phasic alertness is the ability to respond to a stimulus after a warning signal, while selective attention allows the individual to respond to a specific stimulus while ignoring others, and sustained attention is the ability to maintain attention for a period of time^18,20^. All of these components are related to the prefrontal cortex^33,34^, an area that is also negatively affected by sleep deprivation and sleep quality^41^. Poor sleep quality was associated more strongly with a negative effect in cognition than sleep loss^41^. This may be contributing to the worse performance of high school students, since in this group insufficient sleep is accompanied by poor sleep quality^18,32,42^. Another aspect that could explain the worst performance in high school students would be that the cortical functions improve with age because of brain maturation during adolescence, therefore, younger adolescents would be more sensible to sleep loss and poor sleep quality^41,43,44^.

This study has limitations due to the sample size analyzed, which may not have been enough to demonstrate a relation between all variables. Thus, future studies with a greater sample and in different educational institutions can better clarify how sleep habits are affecting cognitive performance of adolescents. Besides, the duration of classes, academic demand, anxiety levels and other mood disorders were not analyzed. These variables would be important to assess how the psychosocial context may be influencing the results observed in this study. Especially with high school students, that usually faces a lot of pressure and stress to get admitted into a good college^45,46^. Another variable that should be analyzed in future studies is chronotype for better understanding of adolescents’ sleep patterns.

In summary, adolescents showed poor sleep quality, sleep irregularity between week and weekend days, signs of sleep deprivation and sleepiness upon awakening irrespective of school level. Thus, these inadequate sleep habits might be related to the effect of school schedules as a temporal challenge for adolescents’ sleep, regardless of educational level. Inadequate sleep may negatively impact their learning and academic achievement. High school adolescents have worse sleep quality and poorer performance on the cognitive task that evaluated attention. This scenario might be due to greater academic demand and psychological pressure from school and parents to high school students to get admitted into a good college. These pressures could lead to higher levels of anxiety impairing the sleep habits and quality of these individuals. As a result, the school performance of adolescents will suffer. Therefore, sleep education programs are recommended in schools for community awareness and additional studies with a larger sample are needed to investigate the causes of these differences in sleep quality and attentional performance between these two educational levels. The results presented can be useful as a way of raising awareness to schools, parents and adolescents, alerting them about the importance of good sleep habits to create a good and healthy learning environment.
